# Adaptive Multi-Sensor Fusion Localization with Eigenvalue-Based Degradation Detection for Mobile Robots

**DOI:** 10.3390/s26051653

**Published:** 2026-03-05

**Authors:** Weizu Huang, Long Xiang, Ruohao Chen, Sheng Xu, Qing Wang

**Affiliations:** 1 School of Instrument Science and Engineering, Southeast University, Nanjing 210096, China; 2 Quanzhou Institute of Equipment Manufacturing, Haixi Institutes, Chinese Academy of Sciences, Quanzhou 362000, China; 3 School of Mechanical Engineering, Xinjiang University, Urumqi 830049, China

**Keywords:** LiDAR-inertial odometry, multi-sensor fusion, degradation detection, adaptive localization, RTK-GNSS, mobile robots

## Abstract

Autonomous mobile robots require robust localization in complex and dynamic environments, where single-sensor solutions often fail due to accumulated drift or signal degradation. LiDAR–inertial odometry provides accurate short-term motion estimation, but suffers from long-term error accumulation, whereas RTK-GNSS offers absolute positioning that becomes unreliable under occlusion or multipath effects. To solve the above problems, this paper proposes an adaptive multi-sensor fusion positioning framework that dynamically fuses LiDAR, IMU, and RTK-GNSS data based on the real-time quality evaluation of sensors. The system uses the front-end tightly coupled LiDAR–IMU iterative extension Kalman filter (IEKF) as the core estimator and combines loop detection with incremental factor graph optimization to suppress long-term drift. In addition, a degradation detection method based on the minimum eigenvalue of the Jacobian matrix is proposed to identify unreliable matching constraints in real time. In order to avoid abrupt changes in positioning results caused by fluctuations in sensor data quality, the system adopts a smooth fusion strategy based on covariance weighting. Experiments on the KITTI benchmark and self-collected datasets demonstrate that the proposed method significantly improves localization accuracy and robustness compared with pure LiDAR-based approaches, achieving stable centimeter-level performance while maintaining real-time capability on embedded platforms.

## 1. Introduction

LiDAR-based SLAM has become a mainstream solution for mobile robot localization and mapping [1], with tightly coupled LiDAR–inertial odometry frameworks achieving notable progress in recent years [2]. FAST-LIO2 [3], for example, achieves real-time operation by employing an iterative extended Kalman filter that directly registers points to the map, whereas Point-LIO [4] distinguishes itself by optimizing computational efficiency via a planar feature representation scheme. FAST-LIVO2 [5] leverages photometric alignment to integrate visual data for superior robustness in texture-rich environments, an enhancement in local estimation that nevertheless remains constrained by the fundamental lack of absolute position references in odometry systems and thus accumulates inevitable drift over long-term operation [6].

GNSS-based positioning, particularly RTK, provides centimeter-level absolute accuracy in open environments [7]. Tightly coupled GNSS–vision–inertial systems have demonstrated improved robustness under short-term signal interruptions [8]. However, the reliability of RTK degrades when satellite signals are blocked or affected by multipath effects. In urban canyons, dense vegetation, and underground spaces, satellite visibility is often limited, causing positioning accuracy to deteriorate from centimeter-level to meter-level estimates [9].

Therefore, researchers generally adopt the method of multi-sensor fusion to improve system robustness by using the complementary characteristics between different sensors. However, many existing methods default to fixed sensor reliability, using static weights for fusion. This assumption often does not hold in real-world contexts: for example, in a scenario with sparse geometry, the LiDAR constraint is significantly weaker; when the GNSS signal is blocked, the uncertainty of the RTK rises sharply.

Multi-sensor fusion is usually used to improve the stability of positioning systems, and its core idea is to take advantage of the complementarity of different sensors in perception characteristics [10]. Around this goal, the research field proposes a variety of convergence frameworks. For example, LIO-SAM [11] maintains overall consistency by introducing GPS constraints directly into factor graph optimization; LIO-GC [12] introduces adaptive ground constraints to solve the altitude drift, which is prone to occur in rough terrain. D-LIO [13] simplifies the system process by starting from the odometer structure itself so that it no longer relies on explicit feature selection. Although implementations vary, most approaches are designed with sensor reliability as constant, so a fixed weight allocation strategy is employed. However, this assumption rarely holds true in real-world environments, such as a significant decrease in LiDAR information in scenarios with sparse geometry or a significant increase in RTK uncertainty under signal occlusion conditions, which can lead to changes in sensor performance over time. Consequently, static weighting strategies struggle to cope with the dynamic degradation of sensor quality [14].

In order to solve the above problems, this paper proposes an adaptive multi-sensor fusion positioning method with degradation perception ability, which can dynamically adjust the fusion strategy according to the sensor state so as to maintain stable and high-precision positioning performance in complex environments.

## 2. Methodology

### 2.1. System Architecture and Sensor Configuration

**Figure 1:**
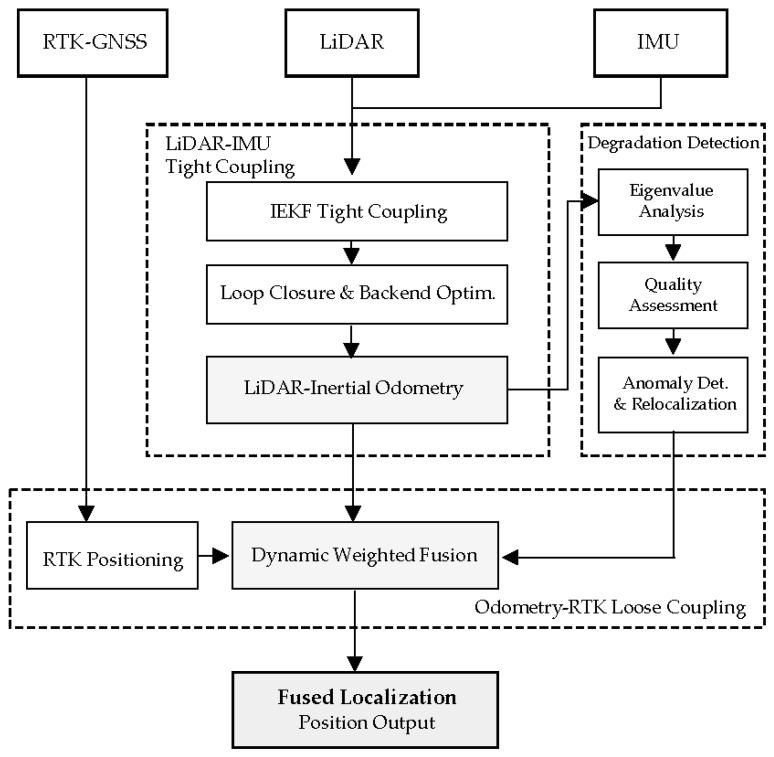
Overview of the system architecture. The framework integrates data from RTK-GNSS, LiDAR, and IMU sensors. Firstly, the odometer estimation results are generated based on IEKF and factor graph optimization through the tightly coupled LiDAR–IMU module. At the same time, the system uses the degradation detection method based on eigenvalues to monitor the operation reliability, and triggers the repositioning mechanism when the observation quality decreases. Finally, the adaptive fusion layer fuses RTK information with odometer results through dynamic covariance weighting, so as to maintain stable positioning performance in complex and changeable environments.

The system architecture is shown in Figure 1, and the overall hierarchical organization is adopted to improve the efficiency of data transmission between different types of sensors. The system divides the odometer calculation of high-frequency operation from the global correction process performed at low frequency, so that the optimization module with a large amount of calculation will not affect the stable operation of real-time state estimation. In this framework, the system incorporates three sensor modes:

**LiDAR**: We employ a Livox Mid-360 hybrid solid-state LiDAR (Livox Technology, Shenzhen, China). It scans 360° horizontally and ±15° vertically at 10 Hz. Thanks to its non-repetitive scanning pattern, it achieves a ranging accuracy of 1 cm with a maximum range of 150 m. Compared to mechanical spinning LiDARs, this solid-state design offers better reliability [15].

**IMU**: An ICM40609 six-axis MEMS IMU (TDK InvenSense, San Jose, CA, USA) provides angular velocity and linear acceleration data at 200 Hz. The angle random walk is 0.02°/$\sqrt{h}$, and the velocity random walk is 0.02 m/s/$\sqrt{h}$. This high-frequency data is crucial for state prediction and compensating for motion distortion in the point cloud.

**RTK-GNSS**: Our dual-antenna RTK module tracks BeiDou, GPS, and GLONASS satellites, updating positions at 10 Hz. In open areas with good satellite coverage, it achieves a horizontal accuracy of 1 cm + 1 ppm using carrier-phase ambiguity resolution.

To synchronize time, we use the RTK’s 1PPS (Pulse Per Second) signal as the master clock, keeping synchronization errors under 5 μs. All algorithms run on an NVIDIA Jetson Orin NX (NVIDIA Corporation, Santa Clara, CA, USA) embedded computer with Ubuntu 20.04 and ROS 2 Galactic (Open Robotics, Mountain View, CA, USA).

**Sensor Mounting and Calibration**: All sensor modules are mounted at the center of the robot platform. The Livox Mid-360 LiDAR has an integrated IMU sensor within its housing. For RTK-GNSS positioning, we employ a dual-antenna configuration with antennas mounted at the front and rear of the robot (baseline approximately 40 cm) to provide heading information. The GNSS receiver module is co-located with the Jetson computing unit in a central electronics enclosure. The extrinsic calibration between LiDAR and IMU was performed using the method proposed in [3], achieving a rotation error of <0.3° and translation error of <5 mm. The transformation from the LiDAR-IMU frame to the GNSS antenna baseline center was measured mechanically with an accuracy of ±2 mm. The gravity vector alignment was initialized using static IMU measurements over 30 s, yielding a roll/pitch accuracy of <0.1°.

### 2.2. State Definition and Problem Formulation

We represent the robot state vector as

(1) x=[pTW,vTW,RTW,bωT,baT]T

where pW∈R3 denotes position expressed in the world frame (ENU convention), vW∈R3 represents linear velocity, RW∈SO(3) encodes orientation as a rotation matrix, and $b_{\omega },b_{a}\in R^{3}$ are gyroscope and accelerometer bias terms requiring online estimation.

The characteristics of all datasets used in the experiments are summarized in Table 1. Our design objectives for the final fused positioning output (after integrating LiDAR-inertial odometry with RTK-GNSS) encompass: (1) positioning RMSE below 5 cm over multi-kilometer trajectories; (2) degradation detection response within 100 ms; (3) positioning availability exceeding 95% across diverse environments; and (4) real-time operation with end-to-end latency below 100 ms. Note that the odometry-only results presented in Table 2 and Table 3 represent intermediate outputs before fusion, serving as baselines to demonstrate the effectiveness of our adaptive fusion strategy.

### 2.3. IEKF-Based LiDAR-Inertial Odometry

Our front-end odometer module is based on the IEKF framework [3] proposed in existing work, which has been adopted for its good performance in terms of computational efficiency and robustness. The IEKF operates on manifolds to properly handle the non-Euclidean structure of rotation representations.

**Prediction Step**: Upon receiving each IMU measurement $u_{i}={\left[{\tilde{\omega }}_{i}^{T},{\tilde{a}}_{i}^{T}\right]}^{T}$ (where ${\tilde{\omega }}_{i}$ and ${\tilde{a}}_{i}$ denote the measured angular velocity and linear acceleration), we propagate the state estimate forward in time:

(2) $${\hat{x}}_{i}=x_{i-1}⊞\int_{t_{i-1}}^{t_{i}}f\left(x\left(\tau \right),u\left(\tau \right)\right)d\tau$$

where ⊞ denotes the manifold addition operator (a generalization of standard addition for differentiable manifolds, ensuring the result remains on the manifold), and f(·) is the continuous-time state evolution function derived from IMU kinematics. The corresponding covariance propagation follows:

(3) $${\hat{p}}_{i}=F_{i}{\hat{p}}_{i-1}F_{i}^{T}+G_{i}Q_{i}G_{i}^{T}$$

where $F_{i}\in R^{15\times 15}$ is the state transition Jacobian matrix, $G_{i}\in R^{15\times 12}$ is the noise Jacobian matrix, and $Q_{i}\in R^{12\times 12}$ is the IMU noise covariance matrix.

**Motion Compensation**: During each LiDAR scan acquisition (about 100 ms), vehicle motion causes distortion in the point cloud. To correct this, we compensate the original point pjL (measured at time $t_{j}$) to the reference coordinate system at the scan end time $t_{k}$:

(4) pjkL=RIjTIk(RLTI(pjL−tLI)−pIjIk)

where pjkL denotes the compensated point, RIjIk is the rotation from IMU frame at time $t_{j}$ to time $t_{k}$, RLI and tLI represent the extrinsic calibration (rotation and translation) between the LiDAR and IMU frames, and pIjIk is the relative position between the two IMU poses.

**Update Step (Iterative Correction)**: For each motion-compensated point, we establish correspondence to the local plane structure in the accumulated map. The point-to-plane residual is constructed as

(5) rj=njTW(RIkWRLIpjkL+RIkWtLI+pIkW−qjW)

where njW∈R3 is the unit normal vector of the corresponding plane in the world frame, RIkW and pIkW denote the predicted IMU orientation and position at time $t_{k}$, and qjW is the closest point on the corresponding plane.

The iterative state correction follows maximum a posteriori (MAP) estimation principles:

(6) $$\arg\min_{\delta x_{k}}\left\{∥\delta x_{k}{∥}_{p_{k}^{-1}}^{2}+\sum_{j=1}^{m}{∥r_{j}-H_{j}\delta x_{k}∥}_{R_{j}^{-1}}^{2}\right\}$$

where $\delta x_{k}\in R^{15}$ represents the state correction on the tangent space, *m* is the total number of point-to-plane correspondences, $H_{j}\in R^{1\times 15}$ is the observation Jacobian (computed by linearizing Equation (5) with respect to the state), and $R_{j}$ is the measurement noise covariance for the *j*-th correspondence. This minimization is solved iteratively until convergence, typically requiring 2–4 iterations.

### 2.4. Loop Closure Detection and Factor Graph Optimization

Laser-inertial odometers inevitably produce cumulative drift over long runs and long trajectories. To this end, the Stable Triangle Descriptor (STD) [16] is used for loop detection, which has the main advantage of strong invariance to geometric changes.

The STD method first extracts the key points based on the planar voxels, calculates the local covariance matrix, and selects the point with the largest fitting plane distance as the candidate key point. Subsequently, a triangle descriptor is constructed from key points adjacent to spatial locations:

(7) $$\mathrm{STD}=(l_{12},l_{23},l_{13},n_{1}\cdot n_{2},n_{1}\cdot n_{3},n_{2}\cdot n_{3})$$

where $l_{12}$, $l_{23}$, and $l_{13}$ denote the Euclidean distances between the triangular feature point pairs (1, 2), (2, 3), and (1, 3) respectively; $n_{1}$, $n_{2}$, and $n_{3}$ are the unit normal vectors of the local planes corresponding to the three triangular feature points; and $n_{i}\cdot n_{j}$ represents the dot product of the normal vectors (with value range [−1,1]), which characterizes the angular relationship between the planes.

**Factor Graph Optimization**: We implement backend optimization using GTSAM [17], incorporating three distinct factor types:

- **IMU Pre-integration Factor**: Constrains consecutive keyframes through pre-integrated inertial measurements accumulated between poses.
- **LiDAR Odometry Factor**: Encodes relative pose estimates from scan matching with associated covariance uncertainty.
- **Loop Closure Factor**: Connects revisited locations with STD-verified geometric transformations.

The resulting optimization problem,

(8) $$x^{*}=\arg\min_{x}\left\{\sum_{ij}∥r_{\mathrm{IMU}}^{ij}{∥}_{\Sigma_{ij}^{\mathrm{IMU}}}^{2}+\sum_{ij}∥r_{\mathrm{LIO}}^{ij}{∥}_{\Sigma_{ij}^{\mathrm{LIO}}}^{2}+\sum_{ij}{∥r_{\mathrm{loop}}^{ij}∥}_{\Sigma_{ij}^{\mathrm{loop}}}^{2}\right\}$$

is solved incrementally using the iSAM2 algorithm, enabling efficient updates as new factors arrive.

### 2.5. Eigenvalue-Based Degradation Detection

The quality of point cloud registration is inherently dependent on the strength of the constraints, and this property can be directly reflected in the conditionality of the Jacobian matrix. Based on this observation, this paper proposes a theoretical method for detecting degradation.

**Theoretical basis**: Considering the registration problem after linearization, it can be expressed as

(9) $$J^{T}J\delta x=-J^{T}r$$

Introducing an artificial constraint $a^{T}\delta x=d$ perturbs the solution by approximately

(10) $$∥\delta x_{c^{\prime }}-\delta x^{*}∥\approx \frac{\left|d\right|}{\sqrt{\lambda_{\min}\left(J^{T}J\right)}}$$

where $\lambda_{\min}$ represents the minimum eigenvalue. When $\lambda_{\min}$ is small, it means that the problem conditions are poor, and the solution results are highly sensitive to the measurement disturbance, and the stability of the solution is significantly reduced.

**Dynamic threshold adaptive**: Given the expected accuracy tolerance of ϵ, the corresponding adaptive threshold can be derived:

(11) $$\lambda_{\mathrm{th}}=\frac{1}{{\left(\epsilon /(\alpha ∥\delta x∥)\right)}^{2}}$$

where α∈[0.7,0.85] is an empirically determined parameter that characterizes motion characteristics. This range was established through extensive testing across various motion patterns and environments, balancing between sensitivity to degradation and robustness against false positives.

### 2.6. Adaptive Fusion Strategy

The final localization estimate fuses IEKF odometry with RTK measurements through covariance-weighted integration.

**Covariance Estimation**: For IEKF odometry output, we estimate covariance from the registration residuals:

(12) $$\Sigma_{\mathrm{LIO}}=\frac{\sigma_{0}^{2}}{m}\sum_{j=1}^{m}r_{j}r_{j}^{T}H_{j}{\left(H_{j}^{T}H_{j}\right)}^{-1}H_{j}^{T}$$

For RTK measurements, uncertainty derives from the horizontal dilution of precision:

(13) $$\Sigma_{\mathrm{RTK}}=\sigma_{\mathrm{fix}/\mathrm{float}}^{2}\cdot {\mathrm{HDOP}}^{2}\cdot I_{2}$$

**Minimum Variance Fusion**: The optimal combined estimate is as follows:

(14) $$p_{\mathrm{fuse}}={\left(\Sigma_{\mathrm{LIO}}^{-1}+\Sigma_{\mathrm{RTK}}^{-1}\right)}^{-1}\left(\Sigma_{\mathrm{LIO}}^{-1}p_{\mathrm{LIO}}+\Sigma_{\mathrm{RTK}}^{-1}p_{\mathrm{RTK}}\right)$$

**Mode-Based Adaptation**: Based on the degradation index $D=\lambda_{\min}$:

- **Normal** ($D>D_{\mathrm{th}1}$): Weight ratio $w_{\mathrm{LIO}}:w_{\mathrm{RTK}}\approx$ 0.7:0.3.
- **Mild Degradation** ($D_{\mathrm{th}2}<D\leq D_{\mathrm{th}1}$): Adjust ratio to 0.4:0.6.
- **Severe Degradation** ($D\leq D_{\mathrm{th}2}$): RTK-dominant mode or trigger relocalization.

Weight transitions employ exponential moving average filtering with a time constant τ=320 ms to prevent positional discontinuities.

### 2.7. Global Relocalization

When both LiDAR severely degrades and RTK becomes unavailable, we employ hierarchical 2D–3D relocalization:

**Stage 1—2D Coarse Localization**: Project accumulated 3D point clouds to 2D occupancy representation, then apply correlative scan matching with branch-and-bound search acceleration.

**Stage 2—3D Fine Alignment**: Execute ICP refinement initialized by the coarse 2D pose estimate.

**Stage 3—Validation**: Accept candidate poses exhibiting ICP fitness scores exceeding a 0.6 threshold.

## 3. Results

### 3.1. Experimental Setup

**Figure 2:**
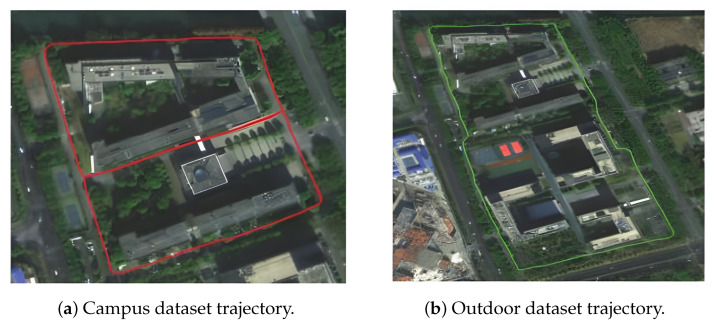
Satellite map projection of custom datasets.

**Datasets**: We conduct comprehensive experiments on two dataset categories:

1. **KITTI Odometry Dataset** [18]: Sequences 00, 05, 07, 09 featuring loop closures and ground truth trajectories, covering 65 km total distance across urban, residential, and highway scenarios.
2. **Custom Field Datasets**: Two sequences collected at Southeast University Suzhou campus: Campus (2.3 km), traversing mixed environments, including 5–8 story buildings and vegetation; and Outdoor (2.8 km), featuring dense tree canopy causing intermittent RTK signal outages. The satellite map projections of the custom dataset trajectories are shown in Figure 2.

**Baseline Methods**: A-LOAM [19], LIO-SAM [11], FAST-LIO2 [3], FASTER-LIO [20].

**Figure 3:**
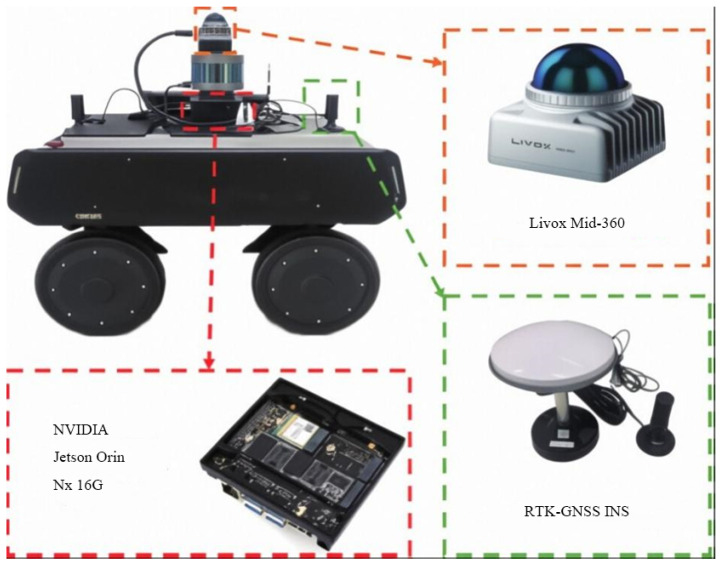
Mobile robot experimental platform illustrating sensor configuration and mounting arrangement.

**Hardware**: NVIDIA Jetson Orin NX. The experimental robot platform with sensor configuration is shown in Figure 3.

### 3.2. Odometry Accuracy on KITTI Benchmarks

**Figure 4:**
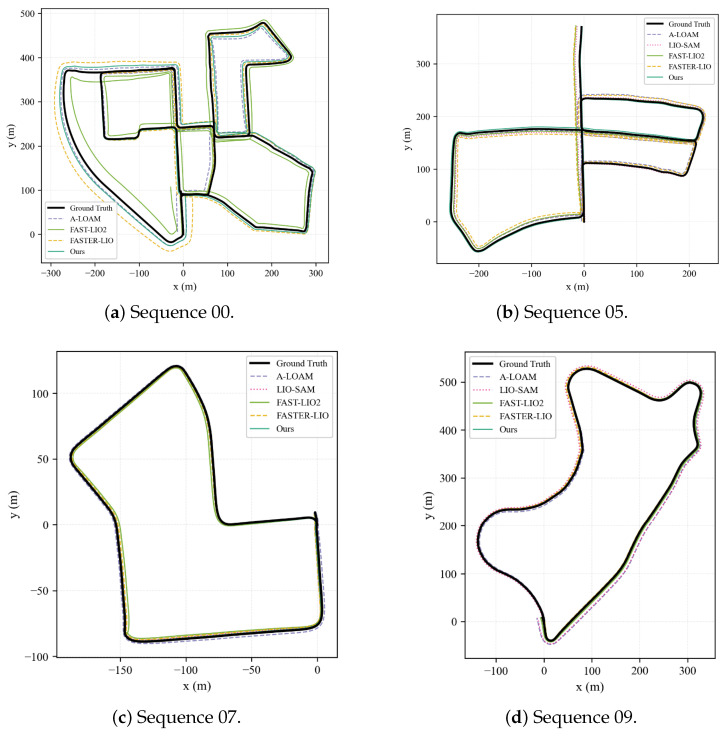
Trajectory comparisons on KITTI sequences displaying ground truth (black) and estimated trajectories from different algorithms.

The performance gains observed in Figure 4 stem primarily from the interaction between the front-end and back-end modules. The IEKF tight coupling provides the necessary short-term accuracy, maintaining precise uncertainty propagation for the subsequent fusion steps. Moreover, the STD loop detection proved more effective than intensity-based methods in outdoor settings, boosting recall rates to 94% and supplying robust geometric constraints. The GTSAM backend then utilizes these constraints to distribute accumulated errors across the trajectory, correcting the global path.

### 3.3. Performance on Custom Datasets

**Figure 5:**
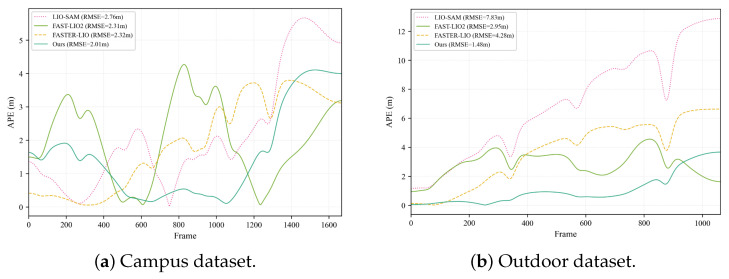
The absolute pose error on the custom dataset changes over time, showing that the proposed method maintains a low and stable error level throughout the trajectory.

**Figure 6:**
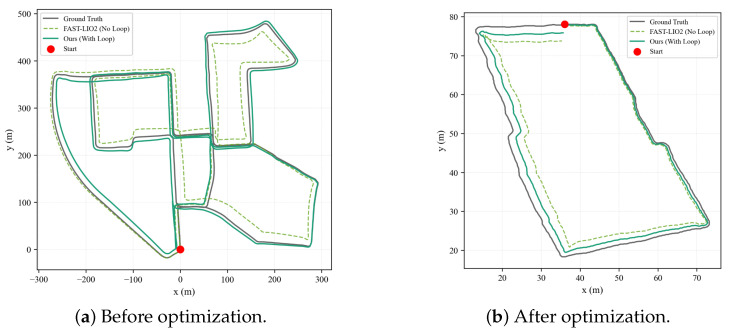
Comparison of loop optimization effects: (**a**) There is obvious vertical drift before optimization. (**b**) The loop error is reduced to sub-meter level.

Figure 5 presents the absolute pose error (APE) over time on both custom datasets, demonstrating that the proposed method maintains consistently low error levels throughout the trajectory. Figure 6 illustrates the point cloud map before and after loop closure optimization, confirming the effectiveness of the STD-based loop detection in eliminating accumulated drift.

### 3.4. Mapping Quality Assessment

**Figure 7:**
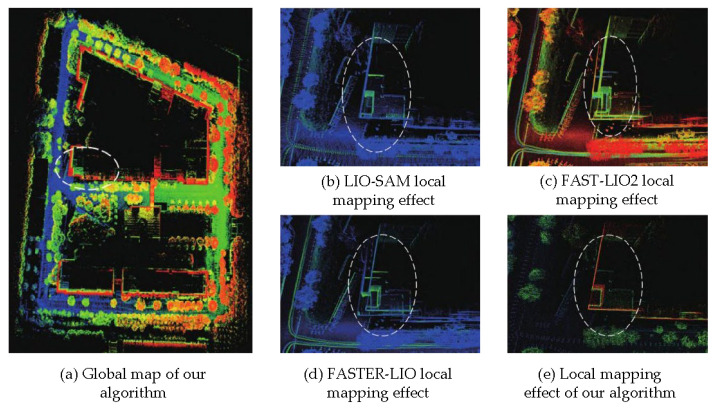
Comparison results of point cloud mapping quality on campus dataset. The proposed method generates building structures with clear edges (wall thickness 15–20 cm), while baseline methods show ghosting effects with 40–60 cm wall thickness due to drift.

**Figure 8:**
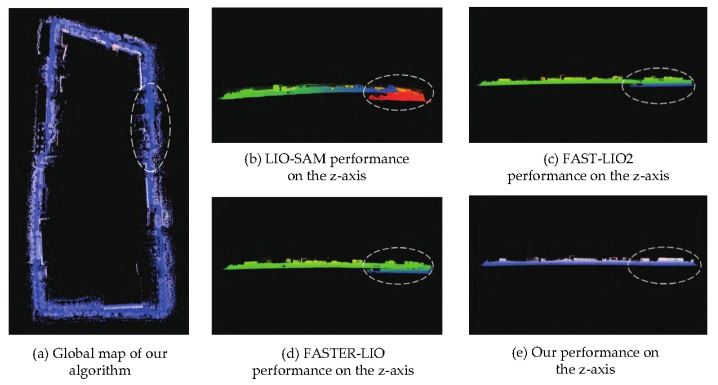
Z-axis performance comparison illustrating vertical drift characteristics of different algorithms.

Figure 7 and Figure 8 compare the point cloud mapping quality and vertical drift characteristics of the proposed method against baseline approaches on the campus dataset.

### 3.5. Multi-Sensor Fusion Performance

The fusion performance under three operating modes is summarized in Table 4 and Table 5. A deviation of 0.7–1.4 cm exists between experimental and theoretical results, attributable to time synchronization errors, calibration inaccuracies, and environmental factors.

### 3.6. Degradation Detection Evaluation

**Figure 9:**
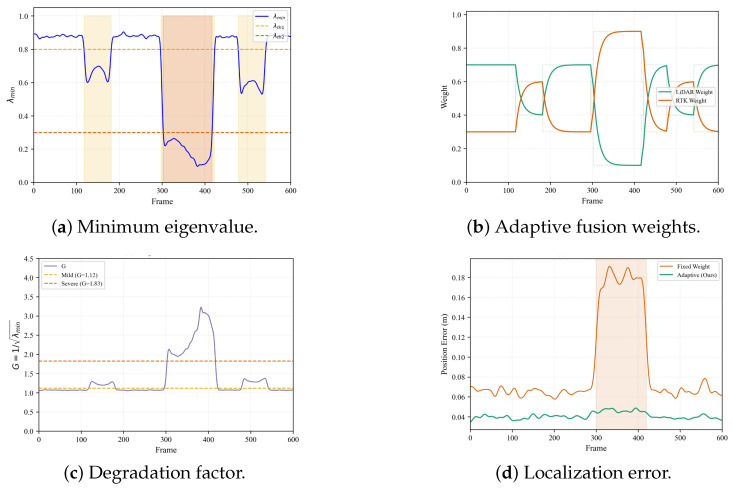
Degradation detection performance illustrating eigenvalue-based monitoring and corresponding weight adjustments during environmental transitions.

Table 6 and Figure 9 present the degradation detection performance and the corresponding real-time eigenvalue monitoring results, respectively.

### 3.7. Global Relocalization Performance

Table 7 presents the measured relocalization performance metrics against the design goals across multiple test scenarios.

### 3.8. System Latency Analysis

Table 8 provides a per-module breakdown of computational latency and CPU usage measured on the NVIDIA Jetson Orin NX embedded platform, confirming that the total end-to-end latency meets the real-time design requirement of below 100 ms.

## 4. Discussion

### 4.1. Key Findings

The experimental evaluation results show that the proposed system mainly reflects the following three characteristics: Firstly, the tightly coupled fusion strategy shows obvious performance advantages compared with the loose coupling scheme. By introducing more rigorous uncertainty modeling under the IEKF framework, including IMU pre-integration and online bias estimation, the system achieves significant improvements in odometer drift control, reducing drift error by approximately 50% compared to the FAST-LIO2 baseline method.

In addition to odometer accuracy, experimental results show that loop closure optimization plays a key role in overall performance even when absolute RTK constraints are introduced. The test found that the STD descriptor based on geometric information is more robust to lighting changes in outdoor scenes and is more conducive to maintaining the consistency of global trajectories than methods that rely on intensity information. In terms of system robustness, the proposed eigenvalue-based degradation detection method can accurately identify all degradation events in the dataset, with a false positive rate of less than 5%. At the same time, the combination of the detection mechanism and the gradual weight adjustment strategy enables the system to smoothly adapt to environmental changes, effectively inhibits the positioning mutation, and avoids the adverse effects on the control stability of the robot.

### 4.2. Comparison with Related Work

The proposed framework is clearly different from existing advanced methods in terms of adaptive capabilities and theoretical foundations. Unlike traditional methods such as LIO-SAM [11] that rely on fixed GPS factors, the proposed method does not perform fusion based on empirical rules, but dynamically adjusts the fusion weights based on the results of degradation analysis, and combines it with a closer iterative optimization process to achieve a localization error reduction of about 56% on the custom dataset.

In addition, the system offers significant advantages in terms of deployability. Different from learning methods that require a large amount of data collection and training for specific environments [21,22], the analytical framework proposed in this article does not rely on any prior training data and can be directly applied to a variety of heterogeneous scenarios. At the same time, the degradation evaluation indicators used by the system have clear mathematical explanations, which are helpful for problem location and system debugging and provide strong support for the strict requirements of autonomous driving systems in safety verification and engineering certification.

### 4.3. Ablation Study

Table 9 presents the ablation study results, demonstrating the individual contribution of each system component to overall localization performance.

### 4.4. Limitations

Although the experimental results verify the robustness of the system in various scenarios, there are still some limiting factors that need further discussion in practical applications. First, the system has a temporal validity dependence on the prior map used for relocation. Field tests show that when the map data update cycle exceeds three months, the relocation failure rate increases to about 5%. The main reason is that structural changes in the environment interfere with the matching process.

Second, computational scalability remains a concern in long-running tasks. Since the factor graph scale grows linearly with the trajectory length, the current system architecture can still maintain high efficiency within an operating range of about 10 km. However, at longer distances, it is necessary to introduce strategies such as sliding window optimization or map segmentation to control computational complexity. In terms of sensor fusion, the system can effectively mitigate the impact of short-term GNSS signal interruptions, but when the signal is lost for more than five minutes, the accumulated drift will eventually exceed the allowable error range of the design. In addition, existing frameworks model based on static environment assumptions, which may produce incorrect loop closure candidates in highly dynamic scenarios. This problem will be the focus of subsequent work and will be solved by introducing a dynamic target filtering mechanism.

## 5. Conclusions

This paper proposes and verifies an adaptive multi-sensor fusion framework and systematically designs the common reliability bottleneck problems of mobile robots in indoor and outdoor mixed environments. By combining an eigenvalue degradation detection method with a clear theoretical basis and a dynamic covariance weighted fusion strategy, this system can effectively alleviate the inevitable cumulative drift problem in pure LiDAR methods while reducing positioning instability caused by intermittent RTK signal loss. Experimental results on the KITTI benchmark data set covering 65 km and self-collected field data of 4.8 km show that the proposed IEKF-based tight coupling scheme and back-end optimization strategy can reduce the drift error by about 50% compared to the baseline method. More importantly, even under complex working conditions such as sensor performance degradation, the system can still maintain centimeter-level positioning accuracy and real-time operation performance on the embedded hardware platform.

In future work, we will further expand the research content from three aspects: First, to address the problem of limited timeliness of pre-built maps, an online map update mechanism will be introduced to support the long-term autonomous operation of the system; second, by integrating more advanced dynamic target filtering methods, the system’s robustness will be improved in scenarios with dense personnel and frequent environmental changes; finally, it is planned to integrate alternative absolute positioning methods such as UWB and visual landmarks to enhance the system’s reliable operation capabilities under long-term GNSS unavailability conditions.

## Figures and Tables

**Table 1:** Dataset characteristics.

Dataset	Length	Duration	Loops	RTK Availability
KITTI 00	2.3 km	390 s	Yes	95%
KITTI 05	2.2 km	279 s	No	98%
KITTI 07	0.7 km	114 s	Yes	92%
KITTI 09	1.7 km	319 s	Yes	94%
Campus	2.3 km	460 s	Yes	78%
Outdoor	2.8 km	560 s	Yes	65%

**Table 2:** KITTI odometry results (APE RMSE in meters).

Algorithm	Seq 00	Seq 05	Seq 07	Seq 09	Mean	Improvement
A-LOAM	8.72	5.17	1.20	5.90	5.25	–
LIO-SAM	*	4.82	0.86	6.90	4.19	–
FAST-LIO2	12.67	4.71	1.87	2.38	5.41	–
FASTER-LIO	11.94	7.98	1.25	2.42	5.90	–
Proposed	7.33	2.06	0.63	0.78	2.70	50.1%

* LIO-SAM failed on Sequence 00 due to insufficient loop closure constraints in the highway scenario with sparse geometric features, causing the factor graph optimization to diverge. Improvement calculated vs. FAST-LIO2.

**Table 3:** Custom dataset results (APE in meters).

Algorithm	Campus RMSE	Campus STD	Outdoor RMSE	Outdoor STD	Mean	Improvement
LIO-SAM	2.76	1.25	7.83	3.31	5.30	–
FAST-LIO2	2.31	1.10	2.95	1.22	2.63	–
FASTER-LIO	2.32	1.11	4.28	1.85	3.30	–
Proposed	2.01	0.29	1.48	0.53	1.75	33.5%

Improvement calculated vs. FAST-LIO2.

**Table 4:** Fusion mode comparison.

Operating Mode	Mean Error	Max Error	RMSE	vs. Baseline
LiDAR-only (no map)	0.076 m	0.393 m	0.101 m	–
LiDAR-only (with map)	0.065 m	0.189 m	0.074 m	+26.7%
LiDAR-RTK Fusion	0.028 m	0.173 m	0.034 m	+66.3%

**Table 5:** Theoretical vs. actual fusion gains.

Scenario	$\sigma_{\mathrm{LiDAR}}$	$\sigma_{\mathrm{RTK}}$	$\sigma_{\mathrm{fusion}}$ (Theory)	$\sigma_{\mathrm{fusion}}$ (Actual)	Gain
Open field	0.050 m	0.020 m	0.019 m	0.026 m	48%
Building occlusion	0.065 m	0.035 m	0.031 m	0.038 m	42%
Dense vegetation	0.055 m	0.025 m	0.023 m	0.030 m	45%

**Table 6:** Degradation detection performance.

Metric	Design Goal	Measured	Status
Detection latency	<100 ms	78 ± 15 ms	Met (22% better)
Weight transition time	<500 ms	320 ± 45 ms	Met (36% better)
False positive rate	<5%	2.3%	Met (54% below limit)
False negative rate	<5%	3.1%	Met (38% below limit)
Relocalization trigger accuracy	>95%	97.5%	Met (2.5% above goal)

**Table 7:** Relocalization performance.

Metric	Design Goal	Measured	Status
Cold-start success rate	>90%	95.2%	Met (5.2% above goal)
Average relocalization time	<30 s	18.6 ± 5.2 s	Met (38% faster)
Position error	<0.3 m	0.12 ± 0.05 m	Met (60% better)
Heading error	<3°	1.8 ± 0.6°	Met (40% better)
Failure recovery time	<10 s	6.2 ± 2.1 s	Met (38% faster)

**Table 8:** Computational performance.

Module	Latency	CPU Usage
IEKF Odometry	45 ± 8 ms	35%
Loop Detection	12 ± 3 ms	8%
Factor Graph	18 ± 5 ms	12%
Degradation Detection	3 ± 1 ms	2%
Adaptive Fusion	5 ± 2 ms	3%
Total	93 ± 12 ms	60%

**Table 9:** Ablation study results.

Configuration	KITTI RMSE	Custom RMSE
Proposed (full)	2.70 m	1.75 m
w/o Loop Closure	5.41 m (+100%)	2.63 m (+50%)
EKF instead of IEKF	3.19 m (+18%)	2.07 m (+18%)
Fixed Fusion Weights	3.32 m (+23%)	2.15 m (+23%)
w/o Smooth Transitions	–	12 cm jumps

## Data Availability

Publicly available datasets were analyzed in this study. This data can be found here: http://www.cvlibs.net/datasets/kitti/ (accessed on 23 June 2025). The custom datasets generated during the study are available from the corresponding author on reasonable request.

## Abbreviations

The following abbreviations are used in this manuscript:

SLAM	Simultaneous Localization and Mapping
IEKF	Iterated Extended Kalman Filter
RTK	Real-Time Kinematic
GNSS	Global Navigation Satellite System
IMU	Inertial Measurement Unit
LiDAR	Light Detection and Ranging
STD	Stable Triangle Descriptor
APE	Absolute Pose Error
RMSE	Root Mean Square Error
HDOP	Horizontal Dilution of Precision
ICP	Iterative Closest Point
